# Dimethylaminomicheliolide (DMAMCL) Suppresses the Proliferation of Glioblastoma Cells via Targeting Pyruvate Kinase 2 (PKM2) and Rewiring Aerobic Glycolysis

**DOI:** 10.3389/fonc.2019.00993

**Published:** 2019-10-02

**Authors:** Jianshuang Guo, Qingqing Xue, Kaihui Liu, Weizhi Ge, Wenjie Liu, Jiyan Wang, Mengyi Zhang, Qiu-ying Li, Dongpo Cai, Changliang Shan, Chunze Zhang, Xinqi Liu, Jing Li

**Affiliations:** ^1^State Key Laboratory of Medicinal Chemical Biology, College of Pharmacy and Tianjin Key Laboratory of Molecular Drug Research, Nankai University, Tianjin, China; ^2^Accendatech Co., Ltd., Tianjin, China; ^3^Department of Colorectal Surgery, Tianjin Union Medical Center, Tianjin, China; ^4^State Key Laboratory of Medicinal Chemical Biology, College of Life Science, Nankai University, Tianjin, China

**Keywords:** dimethylaminomicheliolide, glioblastoma, PKM2 activator, aerobic glycolysis, lactate production

## Abstract

Glioblastoma (GBM) is the most prevalent malignant tumor in the central nervous system. Aerobic glycolysis, featured with elevated glucose consumption and lactate production, confers selective advantages on GBM by utilizing nutrients to support rapid cell proliferation and tumor growth. Pyruvate kinase 2 (PKM2), the last rate-limiting enzyme of glycolysis, is known to regulate aerobic glycolysis, and considered as a novel cancer therapeutic target. Herein, we aim to describe the cellular functions and mechanisms of a small molecular compound dimethylaminomicheliolide (DMAMCL), which has been used in clinical trials for recurrent GBM in Australia. Our results demonstrate that DMAMCL is effective on the inhibition of GBM cell proliferation and colony formation. MCL, the active metabolic form of DMAMCL, selectively binding to monomeric PKM2 and promoting its tetramerization, was also found to improve the pyruvate kinase activity of PKM2 in GBM cells. In addition, non-targeting metabolomics analysis reveals multiple metabolites involved in glycolysis, including lactate and glucose-6-phosphate, are decreased with DMAMCL treatment. The inhibitory effects of DMAMCL are observed to decrease in GBM cells upon PKM2 depletion, further confirming the importance of PKM2 in DMAMCL sensitivity. In conclusion, the activation of PKM2 by DMAMCL results in the rewiring aerobic glycolysis, which consequently suppresses the proliferation of GBM cells. Hence, DMAMCL represents a potential PKM2-targeted therapeutic agent against GBM.

## Introduction

Glioblastoma (GBM) is the most prevalent and malignant primary brain tumor, with a survival rate of 6–9 months after diagnosis (1, 2). Surgical resection followed by concomitant radiotherapy and chemotherapy with the DNA alkylating agent temozolomide (TMZ), is the routine treatment for GBM (3). However, the median survival of treatment with TMZ only increased up to 14.6 months comparing to 12.1 months with radiotherapy alone (2, 4). Therefore, new treatment approaches and novel therapeutic targets are desperately needed for the treatment of GBM (5–7).

The aerobic glycolysis, characterized by increased glucose uptake and lactate production, is a prominent feature of cancer. Global metabolomic profiling on 69 fresh-frozen glioma specimens has revealed that GBMs have accelerated aerobic glycolysis by the accumulation of the glycolytic intermediate phosphoenolpyruvate (PEP) and decrease in pyruvate kinase (PK) activity (8). PK is the last irreversible glycolytic enzyme that catalyzes the reaction of generating pyruvate and ATP from PEP and ADP (9). There are two PKM genes (*PKLR* and *PKM*) and four PK isoforms (L, R, M1, M2) in mammals (9). PKM1 primarily expresses in the brain and muscle while PKM2 specifically expresses in embryonic cells, adult stem cells, and tumor cells. In contrast to the active tetrameric PKM1, PKM2 exists in equilibrium between low activity monomers/dimers and highly active tetramer. This equilibrium is regulated by the allosterically metabolic effectors and post-translational modifications (10). GBM cells were found predominantly express the less active dimeric PKM2, which is key to aerobic glycolysis (11, 12). Thus, small molecule activators capable of promoting tetrameric PKM2 and increasing PK activity could be a potential therapy for GBM (11, 13–15).

**Figure 1:**
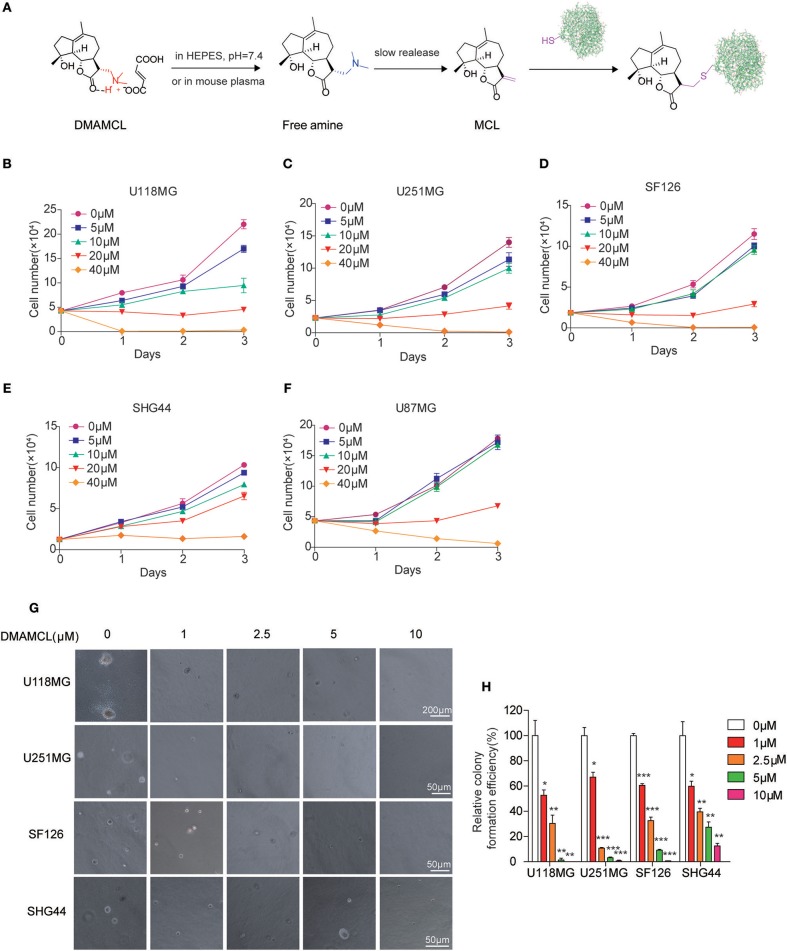
DMAMCL reduces the proliferation of malignant glioma cells and the colony formation through release MCL. **(A)** The release of MCL from DMAMCL. In HEPES buffer, pH = 7.4 or mouse plasma, DMAMCL slowly release MCL. The α-methylene-γ-lactone group of MCL are able to act as a Michael acceptor to form a covalent bond with the thiol group of cysteine residue. **(B–F)** U118MG, U251MG, SF126, SHG44, U87MG cells were grown and treated with DMAMCL at 0–40 μM for 24, 48, and 72 h, respectively. The number of cells was counted. **(G)** Representative malignant glioma colony formation assays from four different cell lines treated with DMAMCL at 0–10 μM for 14 days. **(H)** The statistic of the relative colony formation efficiency. Error bars, mean ± s.e.m., *n* = 3 biological replicates. *P < 0.05, **P < 0.01, ***P < 0.001.

Micheliolide (MCL), a guaianolide sesquiterpene lactone, found in *Michelia compressa* and *Michelia champaca*, exerts selective toxicity against a wide range of tumors (16–21). MCL can alternatively be synthesized from parthenolide (PTL), a traditional natural products with anti-tumor effects in almost all human cancers (22, 23). Compared to PTL, MCL shows comparative anti-cancer activity but remains seven fold more stable in both acidic and basic conditions, making MCL as a promising candidate for cancer therapy (24). Notably, DMAMCL (i.e., ACT001), the fumarate salt form of dimethylamino MCL (Figure 1A), is well-distributed in the brain and is in clinical trials in China and Australia (trial ID: actrn12616000228482) for GBM treatment. However, it remains unclear what MCL/DMAMCL targets in GBM cells and its associated mechanism.

In this study, we verified that DMAMCL inhibited the proliferation and colony formation of various glioma cell lines, especially GBM cells. MCL, the active metabolite in cells, binds PKM2 monomer and increases its PK activity in U118MG cells. The overall metabolomics results showed that DMAMCL dampens the aerobic glycolysis and pentose phosphate pathway, and thus inhibits the cancer cell proliferation. These results suggest that using DMAMCL to improve PKM2 activity could be a potential therapeutic strategy for GBM.

## Materials and Methods

### Chemicals

MCL and DMAMCL were synthesized as previously reported (24). The positive probe (Probe) and the negative probe (NC Probe) were synthesized as previously described (17).

### Reagents

Cell culture reagents including DMEM, trypsin, penicillin/streptomycin, and fetal bovine serum were purchased from Gibco. The silver staining kit (LC6100) was purchased from Thermo Fisher. The pyruvate kinase activity assay kit (MAK072) was purchased from Sigma. Cell counting kit-8 (CCK8) (C6005) was purchased from US Everbright. Lactate colorimetric/fluorometric assay kit (K607-100) was purchased from Biovision. DSS (21555) was purchased from Thermo Scientific. The plasmids of pET28a-PKM2, pET28a-PKM2 C424S, were used as described in Li et al. (17).

### Cell Culture

Human malignant glioma cell lines (U118MG, U251MG, SF126, SHG-44, U87MG) were obtained from ATCC. All cells were cultured in DMEM medium supplemented with 10% (v/v) fetal bovine serum (FBS) in a humidified incubator with 5% CO_2_ at 37°C.

### Cell Viability, Total Cell Number, and Colony Formation Assay

For cell viability assay, human glioma cells were seeded in 96-well-plates (4,000 cells/well). Cells were treated with varying concentrations of DMAMCL for a certain time. 10 μL of CCK8 reagent was added each well and incubated for 1–4 h. Relative cell viability was determined by optical density (OD) values at 450 nm. Total cell number assay was performed by seeding 2 × 10^4^ cells in a 24-well plate and recording cell numbers at the indicated time. For colony formation assay, 1 × 10^3^ cells were plated on 0.3% soft agar, and treated with DMAMCL for 14 days. The numbers of cell colonies were counted under the microscope at 10 × magnification.

### Pull-Down and MS Analysis of MCL-Bound Proteins

The pull-down experiment was carried out following previously described methods (25). Briefly, U118MG cells were plated on a 10 cm tissue culture dish and grown to the confluence for 24 h. Cells were harvested and lysed in RIPA buffer. Probes or NC probes were incubated with cell lysates overnight at 4°C, then the prewashed streptavidin beads (Invitrogen, Carlsbad, CA) were added to each sample and incubates overnight at 4°C. On the second day, the beads were washed six times with RIPA buffer, and the bead-bound proteins were eluted and boiled in SDS loading buffer. The bead-bound proteins were separated by SDS-PAGE and visualized by silver staining. The protein-containing band in the gel was excised, followed by in-gel digestion and analysis by LC-MS/MS (26).

### Western Blot Analysis

Cell lines were lysed in RIPA buffer [50 mM Tris (pH 7.4), 150 mM NaCl, 1% Triton X-100, 1% sodium deoxycholate, 0.1% SDS, and sodium orthovanadate, sodium fluoride, EDTA, leupeptin, etc.] with protease inhibitors and the total protein concentration was quantified with BCA assay (Thermo Fisher Scientific, Waltham, MA). The normalized samples were analyzed by SDS-PAGE and western blot using standard protocols and the following primary antibodies: anti-PKM1 (1:1,000 dilution, sigma, SAB4200094), anti-PKM2 (1:1,000 dilution, Cell Signaling, 4,053), and anti-actin (1:2,000 dilution, Cell Signaling, 4,970), the secondary antibodies: Anti-rabbit IgG, HRP-linked antibody (1:3,000 dilution, Cell Signaling, 7,074). Results are represented as mean and s.e.m. of at least three independent experiments.

### PKM2 Activity Assay

Pyruvate kinase activity was measured as previously described (27). Briefly, for cell line experiments, the medium was replaced with fresh medium 1 h prior to the start of treatment with DMAMCL. Where indicated, 100 μM pervanadate was added 10 min prior to cell lysis. Cells were lysed on ice with NP-40 buffer containing 2 mM DTT and protease inhibitors, then clarified by centrifugation at 12,000 rpm. Pyruvate kinase activity in lysates was determined by the pyruvate kinase activity assay kit (17). Results are represented as mean and s.e.m. of at least three independent experiments.

### Lactate Production Assay

Extracellular lactate production was measured with Lactate Colorimetric/Fluorometric Assay Kit (BioVision).The cells were seeded in a 6-well plate, and after 24 h, cells were treated with DMAMCL for 24 h, the medium was replaced with Opti-MEM and incubated for 1 h at 37°C. After incubation, the medium from each well was assessed using the lactate assay kit. The cell numbers were count for statistical analysis. Results are represented as mean and s.e.m. of at least three independent trials.

### Lentiviral Transduction

The lentiviral transduction was performed as we previously described (17). The shRNA for PKM2 was 5′-CCATAATCGTCCTCACCAA-3′; the shRNA for negative control was 5′-CUUACGCUGAGUACUUCGA-3′ (28, 29).

### RNA Isolation and Quantitative Real-Time PCR for mRNA

The total RNA was extracted by applying TRIzol reagent (Takara) according to the manufacturer's protocol. Immediately after isolation, RNA quantity and quality were determined by using NanoDrop spectrophotometer (Thermo Fisher Scientific). 1,000 ng of total RNA was reversely transcribed using the First Strand cDNA Synthesis kit (Takara). The quantitative PCR analysis was performed in the Real-time Detection System (QuantStudio TM 6) by SYBR Premix Ex Taq II kit (Takara). The sequences of primers are HK2 forward: GAGCCACCACTCACCCTACT, HK2 reverse: CCAGGCATTCGGCAATGTG; GPI forward: CAAGGACCGCTTCAACCACTT, GPI reverse: CCAGGATGGGTGTGTTTGACC; PFK forward: GCACCCTCTCCATTTGATAG, PFK reverse: GCTTATTCCCAGCACACAA; TPI forward: AGTGACTAATGGGGCTTTTACTG, TPI reverse: GCCCAATCAGCTCATCTGACTC; PGK1 forward: TGGACAATGGAGCCAAGTCG, PGK1 reverse: CTCCACTTCTGGGCCTACAC; GAPDH forward: ATCCTGGGCTACACTGAGCA, GAPDH reverse: ATGAGGTCCACCACCCTGTT; ENO forward: AAAGCTGGTGCCGTTGAGAA, ENO reverse: GGTTGTGGTAAACCTCTGCTC; PKM2 forward: ATGTCGAAGCCCCATAGTGAA, PKM2 reverse: TGGGTGGTGAATCAATGTCCA; LDHA forward: ATGGCAACTCTAAAGGATCAGC, LDHA reverse: CCAACCCCAACAACTGTAATCT; β-actin forward: GGAAATCGTGCGTGACAT, β-actin reverse TGCCAATGGTGATGACCT. The PCR amplifications were performed at 95°C for 30 s, followed by 40 cycles of thermal cycling at 95°C for 5 s and 60°C for 45 s.

### Size Exclusion Chromatography

Size exclusion chromatography was performed to isolate PKM2 in different aggregation states. Aliquots of 2 mL of 3 mg/mL purified PKM2 were loaded into a Superdex 200 pg 16/600 column (GE). Two milliliter sample was injected, and the column flow rate was maintained at 1 mL/min. Protein peaks were monitored using the UV absorbance at 280 nm. The retention volumes for the PKM2 peaks were identified by analytical ultracentrifuge. Afterwards, samples were collected for each peak for further PKM2 activity assays. Results are represented as mean and s.e.m. of at least three independent experiments.

### Metabolomics Analysis

For the metabolism measurement, U118MG cells were seed in 10 cm culture dishes to obtain 80–90% confluence. Then the culture was changed to a fresh growth medium containing DMAMCL for 48 h. The cells were harvested and quenched with extraction buffer (methanol:water = 1:1). Prior to GC-MS metabolomics analysis, the metabolites were extracted and derivated. Analysis of metabolites was performed on an Agilent 7890A/5975C GC-MS system (Agilent Technologies Inc., CA, USA). The derivatives were separated using an OPTIMA 5 MS Accent fused-silica capillary column (30 m × 0.25 m × 0.25 μm; MACHEREY-NAGEL, Düren, GER). The peak picking, alignment, deconvolution, and further processing of raw GC-MS data were referred to the previous published protocols (30). The data were normalized against total peak abundances before performing univariate and multivariate statistics. The data was imported to SIMCA software (version 14.1, Umertrics), where multivariate statistical analyses, such as principal component analysis (PCA), partial least-squares-discriminant analysis (PLS-DA), and orthogonal partial least-squares-discriminant analysis (OPLS-DA) were performed. All data were mean-centered and unit variance (UV)–scaled prior to multivariate statistical analysis. The quality of the models is described by the R^2^X or R^2^Y and Q^2^ values. Differential metabolites were determined by the combination of variable importance in the projection (VIP) value (>1) of the OPLS-DA model and the *p*-values (<0.05) from two-tailed student's *t*-tests on normalized peak intensities (31).

### Statistical Analysis

Statistical analysis and graphical presentation were done using GraphPad Prism 5.0. All data are presented as mean ± s.e.m. Results are representative examples of three individual experiments. *P*-values were determined by a two-tailed Student's *t*-test.

## Results

### DMAMCL Suppresses Proliferation and Colony Formation of Various Malignant Glioma Cells

To explore the mechanism of DMAMCL as an anti-cancer agent, we evaluated the growth curve of five glioma cell lines (U118MG, U251MG, U87MG, SF126, SHG44) with DMAMCL treatment. U118MG, U251MG, and U87MG are GBM cell lines, and SF126 and SHG44 are astrocytoma cell lines. The growth of five glioma cell lines was inhibited by DMAMCL treatment in a dose- and time-dependent pattern (Figures 1B–F). The IC_50_ values of DMAMCL against U118MG, U251MG, U87MG, SF126, SHG44 were 17.9, 22.1, 37.1, 25.2, 32.4 μM, respectively at 48 h. Among them, U118MG and U251MG cells were more sensitive and showed around 80 and 70% decreased viability rate with 20 μM DMAMCL for 3 days. Similarly, MCL exhibited the same inhibiting effect at a lower concentration, the IC_50_ values of MCL against U118MG, U251MG, SF126 were 4.2, 14.5, 11.1 μM (Supplementary Figure 1). Furthermore, we investigated the effect of DMAMCL on the oncogenicity of glioma cells by soft agar assay. The results showed that DMAMCL could strongly suppress colony formation in U118MG, U251MG, SF126, SHG44 cells with IC_50_ values at 1.4, 1.2, 1.4, 2.8 μM (Figures 1G,H). These results suggest that DMAMCL markedly inhibits the proliferation of glioma cells. Therefore, GBM cell lines (U118MG and U251MG) were selected for further experiments.

### MCL, the Active Metabolite of DMAMCL, Binds to PKM2 in GBM Cells

**Figure 2:**
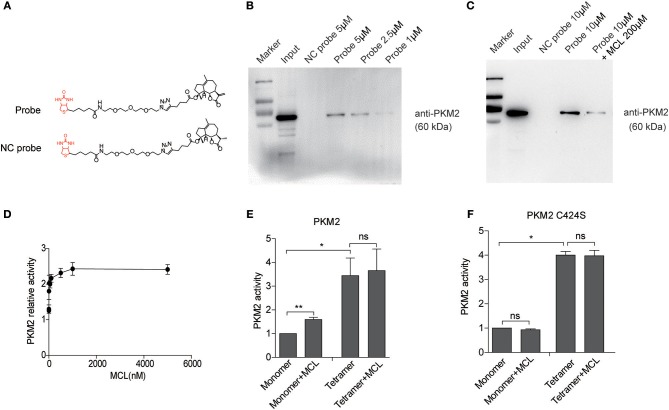
MCL promotes the activity of PKM2 mainly by binds on C424 residue of monomer PKM2. **(A)** Chemical structures of biotin-conjugated MCL (MCL-biotin, Probe) and biotin-conjugated single-bond MCL (MCL-S-biotin, NC probe). **(B)** Western blot detection of PKM2 proteins from probe-cell lysis mixture. **(C)** The U118MG cell lysates were incubated with Probe in the absence or presence of a 20-fold excess of unlabeled MCL overnight at 4°C. PKM2 proteins were detected by western blot. **(D)** Treatment with MCL at a series of concentrations for 90 min, the PKM2 activity was detected. **(E)** The PKM2 activity of the monomer and tetramer was detected treated with 10 μM MCL, respectively. **(F)** PKM2 C424S were purified and the monomer and tetramer were isolated. The activity of PKM2 was determined using the same procedure as above. Error bars, mean ± s.e.m., *n* = 3 biological replicates. *P < 0.05, **P < 0.01, ns, no significant.

Pharmacokinetics studies of DMAMCL revealed that DMAMCL slowly but consistently releases MCL in the plasma and *in vivo* (32). MCL contains a typical α-methylene-γ-lactone group, which can react with accessible cysteine thiol groups to induce modifications of biological functions (33). Using synthesized biotin-conjugated MCL (MCL-biotin, Probe) and inactive probe (MCL-S-biotin, NC probe), we have proved that PKM2 was the primary target of MCL in leukemia cells. To validate whether the target of DMAMCL is PKM2 in GBM cells, we performed pull-down experiments in U118MG cells (Figure 2A) (17). The U118MG-cell lysates were incubated with Probe or NC probe, and the mixture was pulled down with streptavidin-coated agarose beads. The precipitated proteins were resolved by SDS-PAGE and silver staining. As shown in Supplementary Figure 2A, several bands were precipitated by Probe including a distinct band between 55 and 70 kDa. Peptide mass fingerprinting data analysis revealed that 5 peptides of PKM2 were detected in the mass spectrometry (Supplementary Figure 2B), which confirmed PKM2 was the main target of MCL. Immunoblotting was further used to monitor the presence of PKM2 in the precipitates. Consistently, PKM2 was pulled down by Probe, but not NC probe. The signal intensity of PKM2 band increased in a dose-dependent manner of Probe, indicating a strong interaction between Probe and PKM2 in glioma cells (Figure 2B). Moreover, we also observed that an excess of MCL could compete out the PKM2 bound on MCL-Biotin, resulting in the decline of the signal (Figure 2C). Taken together, these results demonstrate that PKM2 is a direct target of DMAMCL in U118MG cells. Since MCL is a multi-target drug, we sought to further verify other potential targets besides PKM2 (data has not been published).

### MCL Activates Monomer PKM2 Through C424 Binding Site in GBM Cells

Our previous work demonstrated MCL activates PKM2 through an irreversible covalent modification on the cysteine 424 residue (C424) of PKM2 in leukemic cells (17). Here, we produced recombinant PKM2 proteins and detected its enzymic activity with the incubation of MCL for 90 min. As shown in Figure 2D, the PK activity was enhanced with the increasing treatment of MCL. It has been reported that recombinant PKM2 (rPKM2, expressed in *E*. *coli*) consists of monomer, dimer, and tetramer (34). In order to know which form of PKM2 bonds to MCL, size-exclusion chromatography and sucrose gradient ultracentrifugation was used to separate rPKM2 into monomers/dimer, tetramer, and polymer (Supplementary Figure 3) (34). The isolated monomers and tetramers were incubated with 10 μM MCL, respectively, and then PK activity was measured. PKM2 tetramers showed higher enzymic activity than their monomeric counterpart. The addition of MCL had minimal effect on tetrameric PKM2 activity, but remarkably increased the activity of monomeric PKM2 by approximately 60% (Figure 2E). In contrast, rPKM2 protein with C424 mutation (PKM2 C424S) did not respond to MCL treatment in either monomeric or tetrameric forms, indicating the dependency of C424 during the MCL-induced PKM2 activation (Figure 2F). In addition, the particle size analysis was carried out to confirm the change of rPKM2, which displayed a significant aggregation of rPKM2 with MCL treatment (Supplementary Figure 4). These results indicate that MCL mainly binds to the monomeric PKM2 at C424 and promotes PK activity of PKM2.

### DMAMCL/MCL Increased Intracellular PK Activity and Decreased Lactate Production in GBM Cells

**Figure 3:**
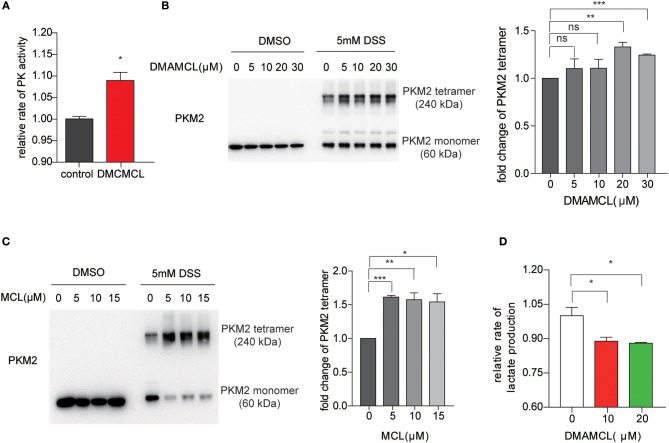
DMAMCL and MCL increase the pyruvate kinase activity by promoting the formation of tetrameric PKM2. **(A)** U118MG cells were incubated with DMAMCL for 24 h, and then the cells were lysed and determined by PK activity assay. **(B,C)** U118MG cells were treated with DMAMCL or MCL for 24 h at indicated concentrations, and then the cell-lysates were crosslinked with DSS. Western blot confirmed the oligomerization of PKM2, and the fold change of PKM2 tetramer was quantified. **(D)** U118MG cells were treated with DMAMCL for 24 h, and the lactate was determined in the DMAMCL-treated medium and control medium. Error bars, mean ± s.e.m., *n* = 3 biological replicates. *P < 0.05, **P < 0.01, ***P < 0.001, ns, no significant.

To investigate whether DMAMCL increases the PK activity in cells, U118MG cells with DMAMCL treatment were assayed for PK activity. Consistent with the MCL results, DMAMCL also significantly increased the PK activity in U118MG cells (Figure 3A). As tetrameric PKM2 is the most active form (35), we sought to determine whether DMAMCL/MCL could increase the formation of tetrameric PKM2 in cells. U118MG cells were incubated with DMAMCL and MCL, respectively, and then the cell lysates were crosslinked with disuccinimidyl suberate (DSS), an uncleavable crosslinker for proteins. The samples were then resolved by SDS-PAGE, and followed by immunoblotting with anti-PKM2. As shown in Figures 3B,C, in the presence of DMAMCL or MCL, the concentration of tetrameric PKM2 significantly increased in accordance with a decrease of monomeric PKM2.

Based on previous studies, tumor cells preferentially express the less-active form of PKM2, which favors lactate production (35). To test whether DMAMCL decreases the following production of lactate, extracellular lactate levels were measured after the treatment of DMAMCL in U118MG cells. Compared with untreated control, lactate levels after treatment of DMAMCL treatment significantly decreased (Figure 3D), indicating that DMAMCL promotes the formation of the active tetrameric PKM2, subsequently decreasing the lactate production in GBM cells.

### DMAMCL Treatment Inhibited Anaerobic Metabolism in GBM Cells

Previous studies have demonstrated PKM2 plays a key role in aerobic glycolysis, and that PKM2 activators could “starve” cancer cells of the biomolecular precursors required to support the rapid cell proliferation associated with metastatic growths (35). To further understand the mechanism of DMAMCL in GBM cells, we interrogated the effects of DMAMCL (10 μM, 48 h) on the cell metabolism. The metabolites (five repeats per treatment) were extracted and analyzed by GC-MS. The R^2^X of the PCA model with 2 principal components was >0.5, indicating a markedly different metabolism between the groups (Supplementary Figure 5A). The R^2^Y and Q^2^ of the PLS-DA model were closer to 1, indicating the model is reliable for explaining and predicting the variations (Supplementary Figure 5B).

**Figure 4:**
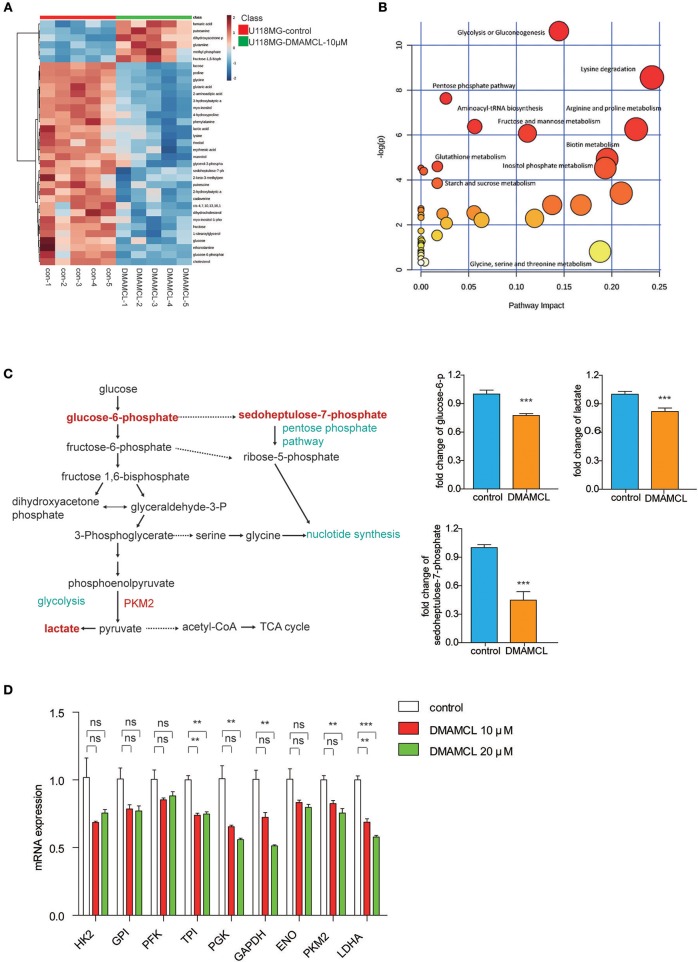
DMAMCL treatment inhibited anaerobic metabolism and anabolism in U118MG cells. **(A)** Heat maps of identified differential metabolites in U118MG cells treated with DMAMCL-10 μM (*n* = 5). **(B)** Differentiated metabolic pathway analysis with MetaboAnalyst 2.0 in U118MG cells treated with DMAMCL-10 μM. **(C)** The glucose metabolism and the key metabolites (glucose-6-P, lactate, sedoheptulose-7-phosphate levels in U118MG cells treated with DMAMCL). **(D)** The mRNA expression of metabolic enzymes involved in aerobic glycolysis. Error bars, mean ± s.e.m., *n* = 3 biological replicates. **P < 0.01, ***P < 0.001, ns, no significant.

The results showed at least 35 metabolites (29 metabolites down-regulated and 6 metabolites up-regulated) that were significantly modified in the group of DMAMCL treatment (Supplementary Table 1). The variations were visualized with heat maps (Figure 4A) and Pearson correlation (Supplementary Figure 6), respectively. Most of the changes can be associated with alterations of the metabolism characteristics of glycolysis, pentose phosphate pathway and amino acid metabolism (Figure 4B). Elevated lactate production and glucose consumption are key features of aerobic glycolysis. As expected, DMAMCL induced a decrease of lactate and glucose-6-phosphate (the first transform of glucose catalyzed by hexokinase) (Figure 4C). In addition, the intracellular concentrations of sedoheptulose-7-phosphate and glycerol-3-phosphate also significantly decreases (Figure 4C). As reported, sedoheptulose-7-phosphate is a key intermediate for the biosynthesis of nucleotides through the pentose phosphate pathway. The glycolysis pathway was most significantly inhibited, to verify the effects of DMAMCL on the aerobic glycolysis enzymes, the U118MG cells were treated with 10 and 20 μM DMAMCL for 48 h, and the mRNA expressions of enzymes including HK2, GPI, PFK, TPI, PGK, GAPDH, ENO, PKM2, LDHA were detected (Figure 4D). Consistent with metabolomics, most metabolic enzymes exhibited a dose-dependent decrease in DMAMCL. Taken together, DMAMCL impairs the survival and proliferation of GBM cells by rewiring aerobic glycolysis.

### Depleted Expression of PKM2 Decreased the Effects of DMAMCL on GBM Cell Viability

**Figure 5:**
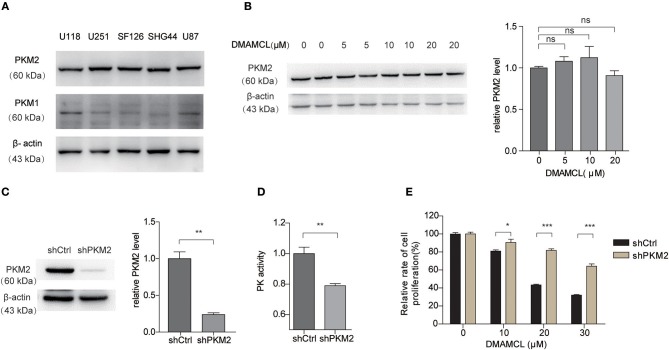
The cell viability inhibition effect of DMAMCL is dependent on the expression of PKM2. **(A)** Western blot detection of PKM2 and PKM1 in different human glioma cell lines. **(B)** The expression of PKM2 with DMAMCL treatment was detected via western blot. **(C)** The depleted expression of PKM2 was confirmed by western blotting. **(D)** The PK activity of U251MG cells was detected with sh*Ctrl* and sh*PKM2* by PK activity assay. **(E)** Depleted expression of PKM2 resulted in a decreased inhibition effect of DMAMCL in U251MG cells after treatment for 48 h. Cell viability was determined by CCK8 assay. Error bars, mean ± s.e.m., *n* = 3 biological replicates. *P < 0.05, **P < 0.01, ***P < 0.001, ns, no significant.

PKM2 was reported to highly express in human glioma samples (14). Consistently, western blot analysis showed that PKM2 highly expressed in five human glioma cell lines (Figure 5A). Basically, DMAMCL did not affect the total protein level of PKM2 in U251MG cells (Figure 5B). In order to verify the importance of PKM2 in the DMAMCL-mediated inhibition of glioma cell viability, we constructed U251MG cell lines stably expressing short hairpin RNA (shRNA) for PKM2 (sh*PKM2*) or control shRNA (sh*Ctrl*). Both PKM2 expression and the PK activity in sh*PKM2* cells were significantly decreased compared to those in *shCtrl* cells (Figures 5C,D). As shown in Figure 5E, after the addition of DMAMCL for 48 h, sh*PKM2* cells were less sensitive to the inhibitory effect of DMAMCL than sh*Ctrl* cells. However, knock-down the expression of PKM2 did not completely abrogate the effect of DMAMCL, which suggested that DMAMCL is a muti-target drug. Taken together, these results proved further evidence that the effect of DMAMCL on GBM cell viability is PKM2-dependent.

**Figure 6:**
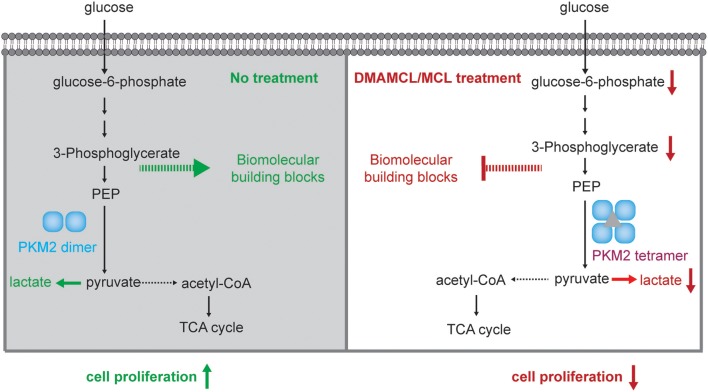
The mechanism of DMAMCL and MCL on GBM inhibition through activating PKM2.

Collectively, these results demonstrated that DMAMCL/MCL inhibited cell proliferation and viability in GBM via direct interaction with PKM2. Promotion of tetramer formation in PKM2 likewise caused a downstream inhibition of anaerobic metabolism and anabolism (Figure 6). This provides effective insight regarding the mechanism for how DMAMCL in GBM treatment functions.

## Discussion

Altered metabolic pathways are one of the hallmarks of cancerous cells (36). Tumor cells mainly rely on aerobic glycolysis (high rates of glucose uptake and lactate production) to produce energy even in the presence of oxygen (10, 37–39). Aerobic glycolysis contributes to cancer cell proliferation by providing the raw components required for to synthesis of cellular materials (40). Increasing evidences have shown that PKM2 plays a critical function in the maintenance of aerobic glycolysis, with decreased PK activity favoring cancer cell proliferation (15, 41, 42). Therefore, targeting PKM2 is a promising therapeutic strategy for the treatment of GBM.

DMAMCL is currently in clinical trials for the treatment of recurrent GBM and has been designated an orphan drug by FDA. Previous studies found a 98.6% inhibition of AML cells in engraft NOD/SCID murine model (24) and significant anti-malignant glioma activities *in vivo* (16). Using leukemic cell HL60, we have proved that MCL, the active metabolite of DMAMCL, binds the C424 residue of PKM2 and to promote PK activity (17).

In this study, we verified that DMAMCL suppressed the cell proliferation and colony formation across different glioma cell lines. GBM cells were found more sensitive to DMAMCL treatment than astrocytoma cells. By using biotin-conjugated probes, we successfully found that MCL directly targeted PKM2, and promoted the subunit association into the active tetramer in GBM cells. The metabonomics profile also demonstrated the majority of the intermediates in glycolysis were reduced, consistent with increased PKM2 activity. Furthermore, depletion of PKM2 decreased the viability inhibition of DMAMCL on GBM cells, demonstrating PKM2 is a key target for DMAMCL in GBM. However, sh*PKM2* cells did not abrogate the whole effect of DMAMCL, which suggests that PKM2 may not be the sole target of DMAMCL. Thus, other targets of DMAMCL needed to be determined.

Recently, using two glioma cell lines (U87-MG and U251), Wang et al. discovered that DMAMCL decreased cell viability and induced apoptosis in glioma cells. Then subsequent experiments showed that the treatment of DMAMCL (40 μM) induced apoptosis and autophagy, which were possibly mediated by ROS generation and Akt/mTOR signaling pathway inhibition (18). The mechanism of drug is directly related to the concentration, incubation time and cell lines etc. Based on our unpublished data, under low concentration (<20 μM), DMAMCL mainly inhibits the cell proliferation, however, under high concentration (20–40 μM), DMAMCL mainly induced apoptosis. In our view, these different mechanisms may due to different concentrations.

In conclusion, DMAMCL is an effective anti-cancer agent for suppressing the proliferation and colony formation in glioma cells, especially in GBM. Our research revealed a novel therapeutic strategy that uses DMAMCL/MCL to activate PKM2 in rewiring the aerobic glycolysis and further inhibit GBM cell growth. This highlights the biological target of DMAMCL for the clinical treatment of GBM.

## Data Availability Statement

All datasets generated for this study are included in the manuscript/Supplementary Files.

## Author Contributions

JG and JL contributed to the conception and design of the experiments. XL, CS, QX, QL, and JL developed the methods. WG synthesized the chemical. JG, QX, KL, WL, JW, and MZ performed the experiments and statistical analysis. JG wrote the paper. JL, MZ, and CS reviewed and revised the paper. DC and CZ provided technical and material support.

### Conflict of Interest

DC was employed by the company Accendatech Co., Ltd, Tianjin, China. The remaining authors declare that the research was conducted in the absence of any commercial or financial relationships that could be construed as a potential conflict of interest.

## Abbreviations

HK2: Hexokinase 2
GPI: Glucose-6-phosphate isomerase
PFK: Phosphofructokinase
TPI: Triosephosphate isomerase
PGK: Phosphoglycerate kinase
GAPDH: Glyceraldehyde-3-phosphate dehydrogenase
ENO: Enolase
PKM2: Pyruvate kinase M2
LDHA: Lactate dehydrohenase A.
