# Supplementary material for: Dimethylaminomicheliolide (DMAMCL) Suppresses the Proliferation of Glioblastoma Cells via Targeting Pyruvate Kinase 2 (PKM2) and Rewiring Aerobic Glycolysis
Source: Front Oncol. 2019 Oct 2;9:993. doi: 10.3389/fonc.2019.00993 (PMC6783512; doi:10.3389/fonc.2019.00993)
Supplement: Supplementary file 1 [file Data_Sheet_1.pdf]

Dimethylaminomicheliolide (DMAMCL) Suppresses the Proliferation of Glioblastoma Cells via Targeting Pyruvate Kinase 2 (PKM2) and Rewiring Aerobic Glycolysis

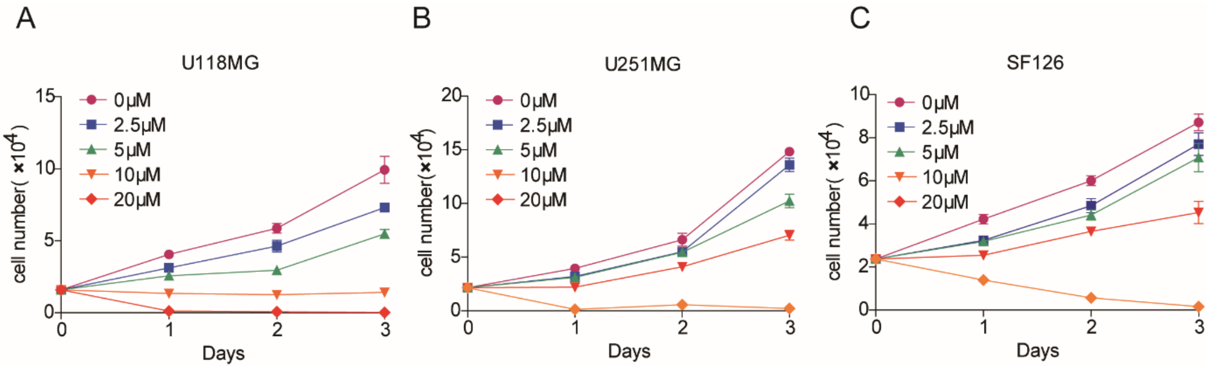

Supplementary Figure. 1. MCL inhibited the proliferation of glioma cells. A-C, U118MG (A), U251MG (B), SF126 (C) cells were grown and treated with MCL at 0-20 μM for 24 h, 48 h, 72 h, respectively by counting cell population.

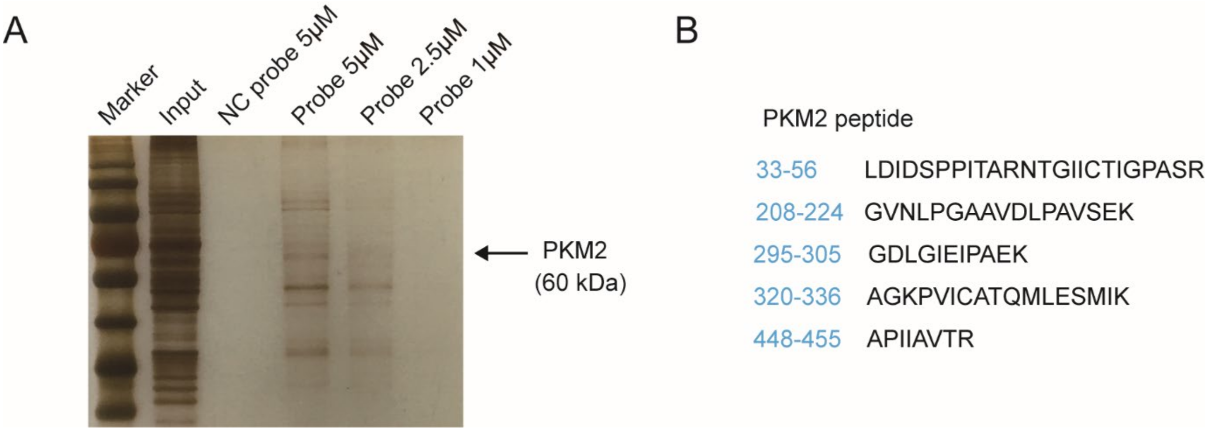

Supplementary Figure. 2. MCL binds PKM2 in U118MG cells. A, The U118MG cells were incubated with either Probe or NC probe at 4 °C overnight. Cell lysates were collected and used for a streptavidin-agarose pull-down assay, and the binding proteins were resolved by SDS-PAGE, followed by silver staining. B, The peptides fragments of PKM2 detected by mass spectrometry**.**

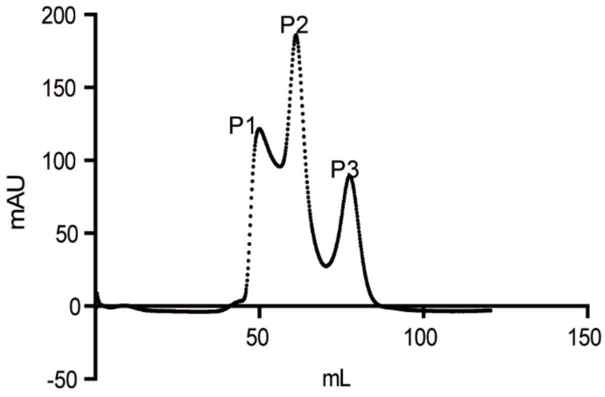

Supplementary Figure. 3. The representative image of size exclusion chromatography. P1 represents polymer, P2 represents tetramer and P3 represents monomer/dimer.

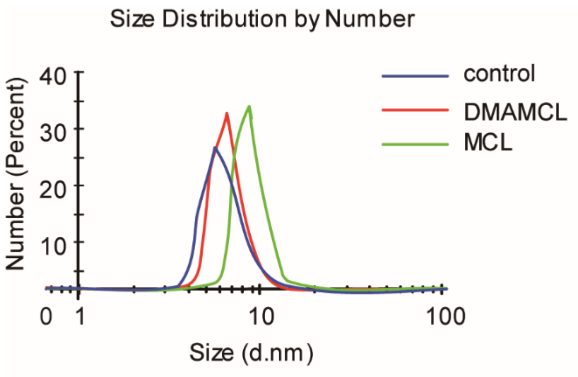

Supplementary Figure. 4. DMAMCL and MCL promote PKM2 aggregation. The purified PKM2 was incubated with DMAMCL, MCL or TEPP46 for 90 min, and then the particle size of PKM2 was determined.

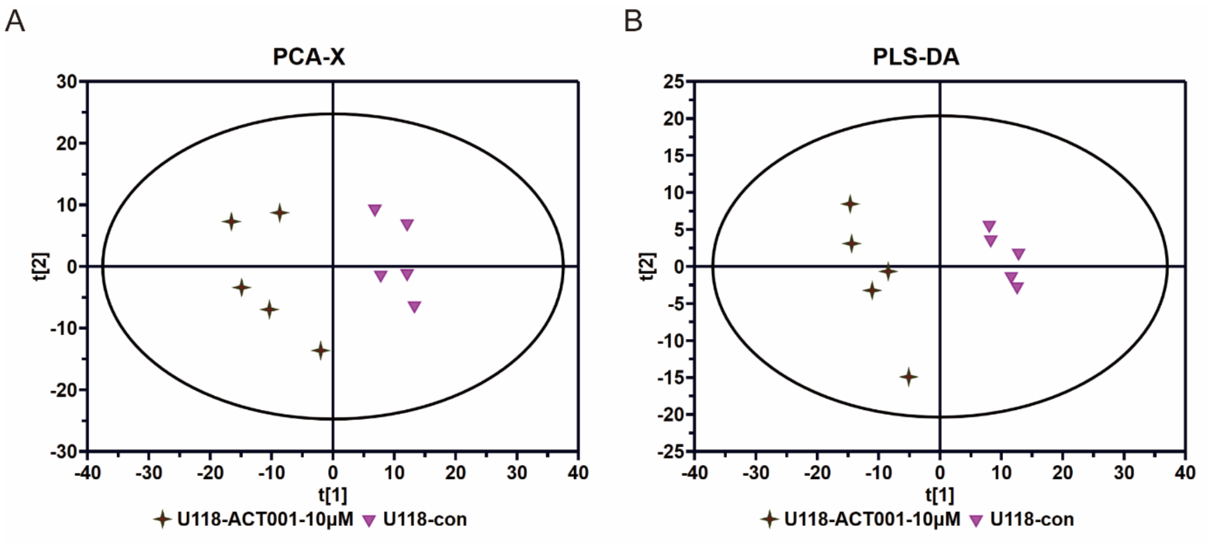

Supplementary Figure. 5. PCA scores plot (A) and PLS-DA scores plot (B) of GC-MS spectra from metabolites in U118MG cells.

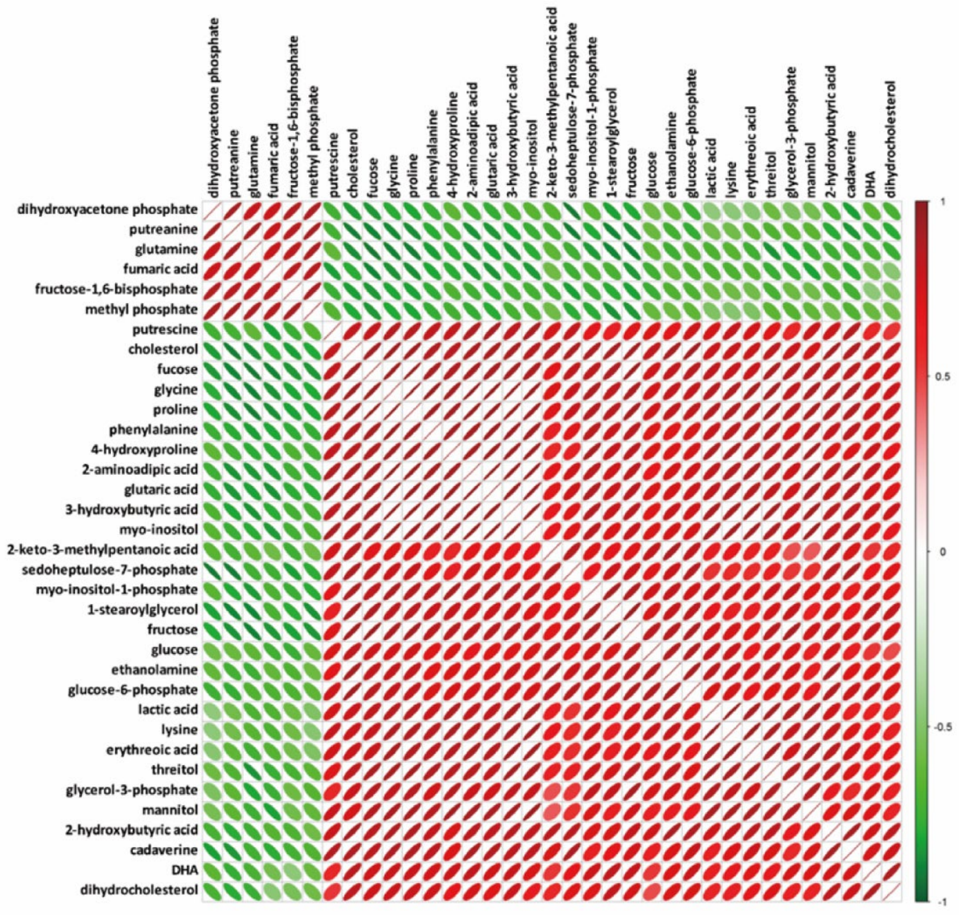

Supplementary Figure. 6. The Pearson correlation of different metabolites between DMAMCL-treated group and control group.

Metabolites	VIP	p-value	FC	rt_mz	DMAMCL					control				
					1	2	3	4	5	1	2	3	4	5
sedoheptulose-7-phosphate	1.40	2.11E-02	-1.16	27.05_387	0.178	0.521	0.660	0.873	0.756	1.335	1.196	1.420	1.303	1.423
2-keto-3-methylpentanoic acid	1.20	4.93E-02	-0.90	9.8_200	0.129	0.228	0.353	0.328	0.346	0.698	0.441	0.560	0.501	0.386
glutaric acid	1.43	3.74E-03	-0.57	14.77_261	0.121	0.118	0.106	0.101	0.109	0.157	0.165	0.184	0.168	0.149
proline	1.49	6.44E-04	-0.56	12.41_142	94.777	90.794	84.713	80.222	85.641	128.469	130.652	127.153	131.377	125.082
fucose	1.48	5.58E-04	-0.53	20.43_117	2.995	2.983	2.605	2.704	2.910	4.102	4.132	3.871	4.128	4.210
ethanolamine	1.30	4.60E-02	-0.46	11.7_174	6.185	6.160	6.651	6.261	6.196	10.589	7.667	8.203	8.399	8.270
2-aminoadipic acid	1.44	1.31E-03	-0.43	20.32_260	10.658	9.830	10.107	9.046	9.531	12.672	13.385	14.216	13.263	12.653
3-hydroxybutyric acid	1.44	4.82E-03	-0.41	9.42_233	0.515	0.465	0.467	0.412	0.436	0.608	0.611	0.617	0.639	0.570
glucose-6-phosphate	1.32	2.44E-02	-0.37	26.38_387	20.959	24.337	22.149	23.045	22.200	32.077	24.903	29.723	28.731	30.022
2-hydroxybutyric acid	1.36	8.73E-03	-0.37	8.69_131	0.264	0.284	0.325	0.261	0.283	0.386	0.338	0.361	0.381	0.361
fructose	1.40	5.21E-03	-0.36	22.36_103	30.497	29.475	25.844	27.600	31.115	39.196	34.894	36.294	36.290	38.887
cis-4,7,10,13,16,19-docosahexaenoic acid	1.21	4.60E-02	-0.34	27.82_91	0.756	0.619	0.676	0.635	0.613	0.823	0.690	0.893	0.860	0.916
1-stearoylglycerol	1.35	1.65E-02	-0.33	28.88_399	0.712	0.618	0.571	0.682	0.713	0.870	0.750	0.815	0.858	0.859
glucose	1.17	4.60E-02	-0.33	22.75_205	41.720	51.620	40.931	41.617	48.237	66.803	52.479	55.586	53.877	52.490
cholesterol	1.46	3.74E-03	-0.30	31.58_329	77.401	76.221	77.077	76.688	79.959	98.927	89.284	96.550	96.656	94.204
lactic acid	1.31	3.73E-02	-0.29	7.35_219	35.587	35.433	32.583	29.104	29.314	43.871	39.829	38.446	39.068	36.478
myo-inositol-1-phosphate	1.34	2.52E-02	-0.28	27_318	10.195	8.710	8.221	8.878	9.598	11.862	10.400	11.588	11.406	10.299
glycine	1.48	6.44E-04	-0.28	12.61_174	418.678	404.670	402.223	379.484	405.323	486.820	487.981	494.788	498.175	473.495
myo-inositol	1.41	5.29E-03	-0.27	24.55_217	117.410	116.374	111.928	103.400	107.303	133.475	137.095	139.339	134.771	125.130
threitol	1.34	1.59E-02	-0.25	16.91_217	8.153	7.780	7.700	6.766	7.592	9.548	9.284	8.447	9.232	8.736
erythreoic acid	1.30	4.60E-02	-0.22	17.57_292	1.885	1.783	1.897	1.553	1.676	2.108	2.077	2.076	2.084	1.922
lysine	1.31	3.74E-02	-0.22	22.83_174	38.232	39.035	37.286	33.016	33.782	44.646	41.946	42.561	42.333	39.113
dihydrocholesterol	1.22	4.93E-02	-0.21	31.67_215	0.316	0.282	0.318	0.298	0.299	0.346	0.319	0.338	0.382	0.367
4-hydroxyproline	1.37	2.11E-02	-0.20	17.24_230	18.323	17.518	16.113	16.165	16.437	19.429	19.161	20.034	19.710	18.589
phenylalanine	1.38	8.24E-03	-0.18	18.98_192	57.733	57.617	54.781	52.613	56.735	61.931	62.982	63.987	66.896	61.791

putrescine	1.34	1.25E-02	-0.18	20.59_174	38.505	42.856	40.460	40.542	39.441	45.025	46.522	48.071	45.816	43.346
cadaverine	1.38	7.31E-03	-0.17	21.84_174	16.466	17.251	18.105	16.828	17.270	18.654	19.123	19.723	19.481	19.469
mannitol	1.34	4.55E-02	-0.16	22.96_319	34.527	33.820	32.109	30.374	30.576	35.654	37.191	36.504	35.589	35.908
glycerol-3-phosphate	1.27	4.93E-02	-0.15	20.92_357	50.074	47.475	44.163	43.944	45.598	53.238	50.626	50.034	49.621	52.292
fumaric acid	1.31	1.28E-02	0.25	13.6_245	8.198	7.647	8.614	8.458	7.705	7.080	6.357	6.666	7.300	6.779
methyl phosphate	1.30	4.60E-02	0.35	9.74_241	97.833	105.468	114.366	96.606	86.271	83.852	74.645	77.085	79.919	77.410
putreanine	1.42	9.13E-03	0.54	24.24_246	1.095	1.236	1.123	1.025	0.982	0.808	0.775	0.713	0.748	0.713
dihydroxyacetone phosphate	1.38	1.36E-02	0.58	20.68_315	17.485	16.062	16.911	13.587	13.642	12.319	10.669	10.595	9.079	9.203
glutamine	1.38	1.32E-02	0.87	21.08_156	20.546	28.969	26.470	25.759	20.731	13.044	11.596	16.364	13.269	12.897
fructose-1,6-bisphosphate	1.32	3.43E-02	0.91	28.51_387	0.914	0.791	0.953	0.757	0.531	0.398	0.330	0.464	0.378	0.528

Supplementary Table 1. The differential metabolites identified between DMAMCL-treated group and control group in U118MG cells.

Abbreviations:

[a]VIP, Variable Importance in the projection, was obtained from the OPLS-DA model.

[b]P-value was analyzed in the " muma " software package in R platform, where parametric tests were performed on the data of normal distribution by Welch's t test, while nonparametric tests were performed on the data of abnormal distribution by Wilcoxon Mann-Whitney test.

[c]FC, fold change, was calculated as a binary logarithm of average mass response (normalized peak area) ratio between Group U118MG-DMAMCL-10 µM vs Group U118MG-con, where a positive value means that the average mass response of the metabolite in Group U118MG-DMAMCL-10 µM is larger than that in Group U118MG-con.
